# Astragalus Granule Prevents Ca^2+^ Current Remodeling in Heart Failure by the Downregulation of CaMKII

**DOI:** 10.1155/2017/7517358

**Published:** 2017-08-10

**Authors:** Sinai Li, Yibing Nong, Qun Gao, Jing Liu, Yan Li, Xiaoyun Cui, Jie Wan, Jinjin Lu, Mingjie Sun, Qian Wu, Xiaolu Shi, Haifeng Cui, Weihong Liu, Mingxue Zhou, Lina Li, Qian Lin

**Affiliations:** ^1^Dongfang Hospital, Beijing University of Chinese Medicine, Beijing 100078, China; ^2^Beijing Hospital of Traditional Chinese Medicine, Capital Medical University, Beijing Institute of Traditional Chinese Medicine, Beijing 100010, China; ^3^Department of Medicine, Institute of Molecular Cardiology, University of Louisville, Louisville, KY 40292, USA; ^4^Experimental Research Center, China Academy of Chinese Medical Sciences, Beijing 100700, China; ^5^College of Basic Medicine, Beijing University of Chinese Medicine, Beijing 100029, China

## Abstract

**Background:**

* Astragalus* was broadly used for treating heart failure (HF) and arrhythmias in East Asia for thousands of years. Astragalus granule (AG), extracted from* Astragalus*, shows beneficial effect on the treatment of HF in clinical research. We hypothesized that administration of AG prevents the remodeling of L-type Ca^2+^ current (*I*_Ca-L_) in HF mice by the downregulation of Ca^2+^/calmodulin-dependent protein kinase II (CaMKII).

**Methods:**

HF mice were induced by thoracic aortic constriction (TAC). After 4 weeks of AG treatment, cardiac function and QT interval were evaluated. Single cardiac ventricular myocyte was then isolated and whole-cell patch clamp was used to record action potential (AP) and *I*_Ca-L_. The expressions of L-type calcium channel alpha 1C subunit (Cav1.2), CaMKII, and phosphorylated protein kinase A (p-PKA) were examined by western blot.

**Results:**

The failing heart manifested distinct electrical remodeling including prolonged repolarization time and altered *I*_Ca-L_ kinetics. AG treatment attenuated this electrical remodeling, supported by AG-related shortened repolarization time, decreased peak *I*_Ca-L_, accelerated *I*_Ca-L_ inactivation, and positive frequency-dependent *I*_Ca-L_ facilitation. In addition, AG treatment suppressed the overexpression of CaMKII, but not p-PKA, in the failing heart.

**Conclusion:**

AG treatment protected the failing heart against electrical remodeling and *I*_Ca-L_ remodeling by downregulating CaMKII.

## 1. Introduction

Heart failure (HF) remains one of the leading factors of human mortality in the western world [1]. Patients with HF are at an increased risk of malignant arrhythmia, accounting for a substantial component of mortality associated with this disease [2]. HF modifies the operation of ion channels and transporters in a way that promotes the occurrence of cardiac rhythm disturbances, a process called “electrical remodeling [3].”* Astragalus* was broadly used for treating HF and arrhythmias in East Asia for thousands of years. The cardioprotective effects of the ingredients from* Astragalus* have been confirmed in pharmacology studies [4–8]. Astragalus granule (AG), extracted from* Astragalus*, shows beneficial effect on the treatment of HF in clinical research [9]. However, its effects on HF-associated electrical remodeling have not been addressed. The present study provided evidence supporting the effectiveness of AG in protecting the failing heart against electrical remodeling.

## 2. Materials and Methods

### 2.1. Animals

Male C57BL/6N mice, 6–8 weeks old, were purchased from Beijing Vital River Animal Technique Limited Corporation (Beijing, China). The mice were housed in cages at 22 ± 2°C, humidity 40 ± 5%, under a 12-hour light/dark cycle with standard diet and water ad libitum. Experiments were started after the animals acclimating for a week.

### 2.2. Ethics Statements

All animal experiments were carried out in accordance with the European Union adopted Directive 2010/63/EU. The experiment protocols were approved by the Joint Ethical Review Committee of Beijing University of Chinese Medicine according to the Regulation of Experimental Animal Management (State Scientific and Technological Commission of the People's Republic of China, number 2, 1988).

### 2.3. Drugs

AG (batch number 15014601) was obtained from Beijing Tcmages Pharmaceutical Limited Corporation (Beijing, China), which contained chiefly of 1.48 mg/g Astragaloside IV and 0.90 mg/g calycosin-7-glucoside. The quality control of AG was shown on the high-performance liquid chromatography (HPLC) images in Supplementary Material available online at https://doi.org/10.1155/2017/7517358.

### 2.4. Solutions

Ca^2+^-free Tyrode solution for cell isolation contained 148.8 mM NaCl, 4 mM KCl, 0.53 mM MgCl_2_, 5 mM 4-(2-hydroxyethyl)-1-piperazineethanesulfonic acid (HEPES), 5 mM D-glucose, and 0.33 mM NaH_2_PO_4_, with pH adjusted to 7.4 with NaOH. Kraft-Bruhe (KB) solution for storage of cells contained 120 mM KOH, 120 mM L-glutamic acid, 5 mM MgCl_2_, 20 mM taurine, 1 mM ethylene glycol tetra-acetic acid (EGTA), 10 mM HEPES, and 10 mM D-glucose, with pH adjusted to 7.4 with KOH. The test solution for AP recording was Tyrode's solution with 1.8 mM Ca^2+^. The pipette solution for AP recording contained 10 mM NaCl, 120 mM KCl, 20 mM HEPES, 5 mM EGTA, and 3 mM MgATP, with pH adjusted to 7.2 with KOH. The test solution for *I*_Ca-L_ recording contained 137 mM NaCl, 120 mM KCl, 20 mM HEPES, 5 mM EGTA, and 3 mM MgATP, with pH adjusted to 7.4 with NaOH. The pipette solution for *I*_Ca-L_ recording contained 140 mM CsCl, 4 mM MgCl_2_, 10 mM HEPES, 10 mM EGTA, and 4 mM Na_2_ATP, with pH adjusted to 7.3 with CsOH.

### 2.5. Animal Model and Drug Administration

The mice were randomly assigned into three groups: thoracic aortic constriction (TAC) (*n* = 12), TAC + AG (*n* = 12), and sham groups (*n* = 12). The procedure of TAC has been well described previously [10]. Briefly, the mice were anesthetized with chloral hydrate (400 mg/kg intraperitoneally) after fasting for 12 h. The mice were then orally intubated with 20-gauge tubing and ventilated (Kent Scientific Co., CT, USA) at 120 breaths/min (0.1 mL of tidal volume). A 3 mm left-sided thoracotomy was created at the second intercostal space. The transverse aortic arch was ligated (7-0 Prolene) between the innominate and left common carotid arteries with an overlying 28-gauge needle, and then the needle was removed, leaving a discrete region of stenosis. The chest was closed, and the left-sided pneumothorax was evacuated. Perioperative (24 h) mortality was less than 10%. The animals in the sham group underwent the same procedure but without constriction. The mice in the TAC and sham groups received 0.5 mL of saline daily by gavage for 4 weeks. The mice in the TAC + AG group received AG solution (100 mg in 0.5 ml saline) daily by gavage for 4 weeks.

### 2.6. Echocardiographic Analysis

Echocardiography was performed using a Vevo 770 ultrasound system (Visualsonics, Toronto, Canada) equipped with a real-time microvisualization scan head probe (RMV-707B) working at a central frequency of 30 MHz. Mice were anesthetized with isoflurane, at a concentration of 4% (induction) and 1.5% (maintenance) in 100% oxygen. Each animal was placed on a heating table (32 degrees Celsius) in a supine position with the extremities tied to the table through four electrocardiography leads. The chest was shaved using a chemical hair remover (Nair, Church & Dwight Co., NJ, USA). Ultrasound gel was applied to the thorax surface to optimize the visibility of the cardiac chambers. The heart rate (HR) of the animals was recorded simultaneously. Echoes were acquired at week 4 after TAC. The left ventricle (LV) systolic function was evaluated by the ejection fraction (EF) and fractional shortening (FS) of the LV. EF and FS were calculated from the LV diameters (LVIDd, LVIDs) measured on an M-mode examination of the LV. To obtain the classical LV M-mode tracing, the M-mode cursor was vertically positioned at a transthoracic parasternal short axis view (visualizing both papillary muscles). The LV diastolic function was evaluated using pulsed-wave Doppler or tissue Doppler from LV apical four chamber views. The early diastolic mitral inflow velocity (*E*) and early diastolic mitral annular velocity (*E*_*m*_) were measured from the pulsed- wave Doppler graph and the tissue Doppler graph, respectively. The ratio of *E*/*E*_*m*_ was calculated as an indicator of LV diastolic function. Measurements were performed according to the American Society for Echocardiography and were acquired at a heart rate of 400–450 beats per minute (bpm). At least three measurements were taken and averaged for each parameter.

### 2.7. Electrocardiogram Recordings and Analysis

All electrocardiogram (ECG) recording sessions were performed during daytime. Six-lead restrained ECG monitoring of conscious mice was performed with the ecgTUNNEL system platform (EMKA Technologies, France). The animals were allowed to stay in the restraining system for 5 min before starting ECG recordings to minimize the effects of stress. Six leads of ECG were recorded for 20 min using the IOX software (EMKA Technologies). Lead II recordings were analyzed using the Ecg-autosoftware (EMKA Technologies). QT intervals were measured as the time interval between the initiation of the QRS complex and the end of the T-wave, and corrected QT intervals (QTc) were calculated using the formula: QTc = QT/(RR/100)^1/2^.

### 2.8. Cardiac Ventricular Myocyte Isolation

Single cardiac ventricular myocyte was isolated from the heart of mice as previously described with slight modifications [11]. Briefly, 5 min after heparinization (1000 U/kg intraperitoneally), the animals were anesthetized with chloral hydrate (400 mg/kg intraperitoneally). The heart was rapidly excised, mounted on the Langendorff apparatus, and perfused via the aorta with Ca^2+^-free Tyrode solution for 5 min and then with Ca^2+^-free Tyrode solution containing 0.6 mg/mL collagenase II (Worthington Biochemical Co., NJ, USA) for 15–20 min at 37°C. Next, the left ventricular wall was cut, put into a culture dish filled with KB solution, and blown gently to obtain single ventricular myocytes. All the steps were performed at 37°C in solutions gassed with 95% O_2_ and 5% CO_2_. The cells were maintained at room temperature in KB solution until use. Only calcium-tolerant, quiescent, and rod-shaped cells showing clear cross-striations were studied.

### 2.9. Electrophysiological Recordings

Isolated myocytes were investigated in a continuously superfused (1.5 mL/min) recording chamber fixed to an inverted microscope. The whole-cell patch clamp technique was used to record AP and *I*_Ca-L_ using an EPC10 amplifier with the Patchmaster software (HEKA Electronics, Germany). Borosilicate glass patch pipettes (resistance = 3–5 MΩ) were pulled using a P-97 Micropipette Puller (Sutter Instrument Company, CA, USA). The junctional potential was corrected by zeroing the potential just before the pipette tip touched the cell membrane. After the cell membrane was broken by applying additional suction, cell capacitance and series resistance were electrically compensated. After access was gained in the whole-cell voltage clamp configuration, the myocytes were allowed to equilibrate for 5 min with the internal solution before the data were collected. All of the recordings were performed at room temperature (22°C) within 30 min to avoid current rundown. The action potential (AP) of myocytes was recorded by applying a 900 pA current pulse with duration of 3 ms at 0.2 Hz in the current-clamp mode. The *I*_Ca-L_ traces were obtained during a depolarizing pulse from the holding potential of −40 mV to +50 mV at 0.1 Hz in 10 mV increments for 300 ms. Current amplitudes were expressed as current density by dividing the absolute current by cell capacitance (pA/pF) to correct for variability in cell size. *I*_Ca-L_ inactivation was modeled as a biexponential decay, and mean values of fast (*τ*1) and slow (*τ*2) time constants were calculated.

### 2.10. Western Blot Analysis

The mice were sacrificed after 4 weeks of drug intervention, and 50 mg of myocardial tissue was sampled from the left ventricle and stored at –80°C. The whole protein of the tissues was extracted with a protein extraction kit (C1053; Applygen Technologies, Beijing, China), according to the manufacturer's instruction. The concentration of the whole protein was determined with a bicinchoninic acid protein assay kit (P1511; Applygen Technologies, Beijing, China). The whole protein was mixed with electrophoresis sample buffer. After separating on 10% sodium dodecyl sulfate-polyacrylamide gel electrophoresis gel, the proteins were transferred onto polyvinylidene difluoride membranes. After 1 h blocking with 5% nonfat dry milk and rinsing with Tris-buffered saline-Tween 20 (TBS-T) thrice (5 min each), the membrane with target proteins was cut and incubated overnight at 4°C with the following antibodies: anti-CaMKII antibody (ab52476; Abcam, UK), anti-Cav1.2 antibody (ab84814; Abcam), anti-PKA (phosphoT197) antibody (ab75991, Abcam), and anti-GAPDH antibody (TA-08; ZSbio, China). Afterward, the membranes were rinsed thrice (5 min each), incubated with secondary antibody for 1 h at room temperature, and rinsed with TBS-T thrice, 5 min each time. The protein was quantified by scanning densitometry in the X-ray film using a bioimage analysis system. The result was expressed as a relative optical density compared with that from GAPDH.

### 2.11. Statistical Analyses

All continuous variables are presented as the mean ± standard error (SE) and all categorical variables are presented as number (percent) of animals in each group. All continuous variables were analyzed with one-way ANOVA for normally distributed data or Kruskal-Wallis one-way analysis of variance on ranks for data that are not normally distributed. Kaplan-Meier analysis was implemented to analyze cumulative overall survival. The Prism 6 software and SPSS 16.0 statistical software were used for data analysis and graphic design. Differences with *P* values less than 0.05 were considered statistically significant.

## 3. Results

### 3.1. Survival

**Figure 1:**
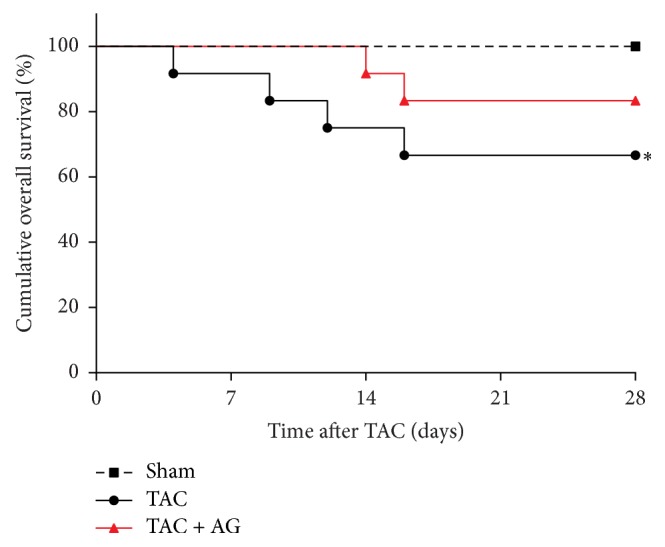
Cumulative overall survival. Kaplan-Meier survival curve from groups. ^*∗*^*P* < 0.05 versus sham group.

All mice in the sham group survived during 4 weeks of observation, while 4 of 12 mice in the TAC group died of heart failure, representing a survival rate of 66.7%. Treatment with AG showed a trend to restore the survival rate of mice, reaching 83.3% at the end of the experiment with two mice dying of heart failure (Figure 1). Using log-rank test of Kaplan-Meier analysis, the survival rate between sham and TAC group was statistically significant (*P* = 0.032), while the rate between TAC and TAC + AG group was not (*P* = 0.306).

### 3.2. Effect of AG on Heart Function

**Figure 2:**
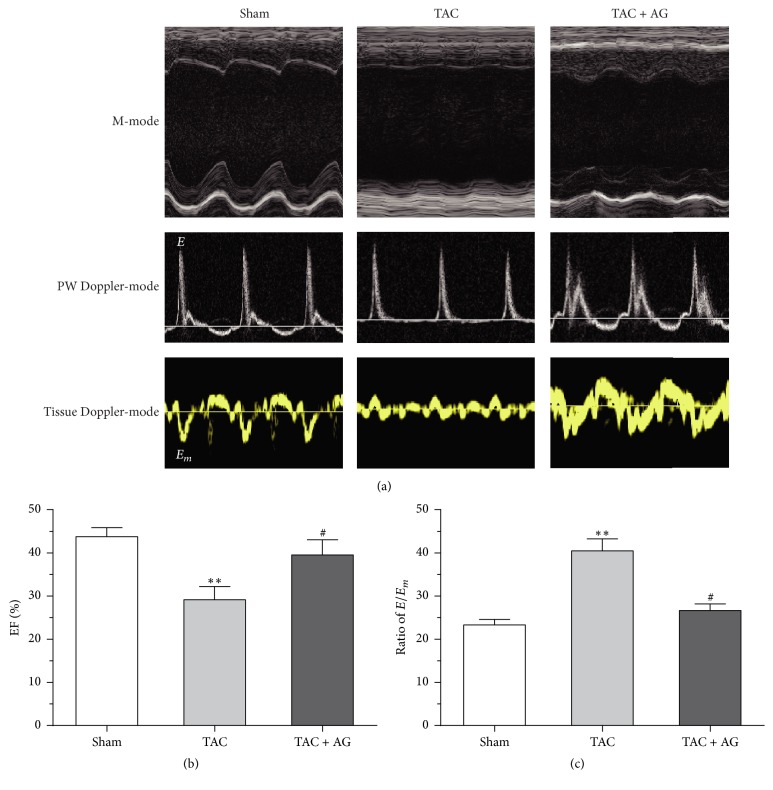
Effect of AG on heart function. (a) Representative echocardiography images from groups. (b) The left ventricle ejection fraction from the sham (*n* = 12), TAC (*n* = 8), and TAC + AG (*n* = 10) groups. (c) The ratio of *E*/*E*_*m*_ from the sham (*n* = 12), TAC (*n* = 8), and TAC + AG (*n* = 10) groups. ^*∗∗*^*P* < 0.01 versus sham group, ^#^*P* < 0.05 versus TAC group.

Four weeks after the TAC surgery, echocardiography showed that the cardiac systolic and diastolic functions of mice in the TAC group were impaired. The EF in the TAC group decreased compared with the sham group (29.16 ± 3.03% versus 43.73 ± 2.11%, *P* < 0.01); this decrease was accompanied by an increase in *E*/*E*_*m*_ (40.44 ± 2.80 versus 23.29 ± 1.30, *P* < 0.01). These alterations were ameliorated by administering AG. After treatment with AG for 4 weeks, the EF apparently increased in the TAC + AG group (39.51 ± 3.53% versus 29.16 ± 3.03%, *P* < 0.05), accompanied by decreased *E*/*E*_*m*_ (26.66 ± 1.50 versus 40.44 ± 2.80, *P* < 0.05), compared with the TAC group (Figure 2 and Table 1 in Supplementary Material).

### 3.3. Effect of AG on Electrophysiology in Heart Failure

**Figure 3:**
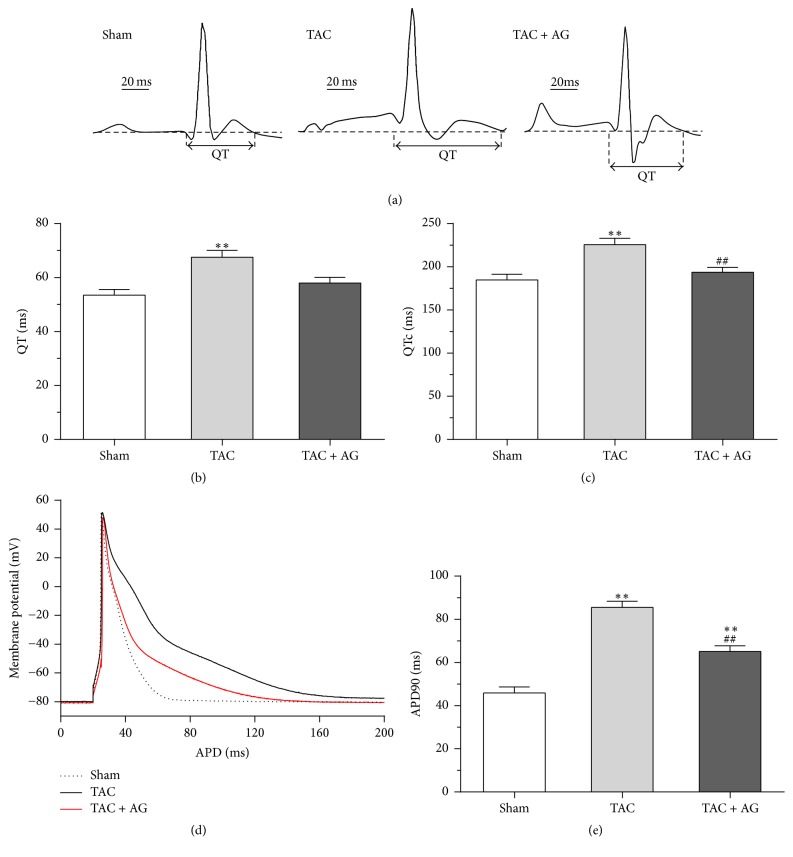
Effect of AG on electrophysiology in heart failure. (a) Representative ECG images from the sham, TAC, and TAC + AG groups. (b) The QT from the sham (*n* = 12), TAC (*n* = 8), and TAC + AG (*n* = 10) groups. (c) The QTc from the sham (*n* = 12), TAC (*n* = 8), and TAC + AG (*n* = 10) groups. (d) Representative AP traces from groups. (e) The APD90 from each group (*n* = 6). ^*∗∗*^*P* < 0.01 versus sham group, ^##^*P* < 0.01 versus TAC group.

ECG images were acquired by a six-lead restrained ECG monitor in each group (Figure 3(a)). QT (66.71 ± 2.82 ms versus 53.45 ± 2.11 ms, *P* < 0.01) and QTc (223.80 ± 8.10 ms versus 184.66 ± 6.62 ms, *P* < 0.01) were apparently prolonged in the TAC group compared with the sham group. In contrast, QT (57.92 ± 2.12 ms versus 66.71 ± 2.82 ms, *P* = 0.067) and QTc (193.59 ± 5.81 ms versus 223.80 ± 8.10 ms, *P* < 0.01) in the TAC + AG group were shortened compared with those in the TAC group, indicating that AG treatment for 4 weeks obviously attenuated TAC-induced increase in QT interval (Figures 3(b) and 3(c)).

The action potential duration obtained with 90% repolarization (APD_90_) was recorded. The APD_90_ was significantly prolonged in the TAC group (85.47 ± 2.86 ms versus 45.88 ± 2.77 ms, *P* < 0.01) compared with the sham group. Compared with the TAC group, APD_90_ of TAC + AG group apparently was shortened (65.03 ± 2.62 ms versus 85.47 ± 2.86 ms, *P* < 0.01) (Figures 3(d) and 3(e)). This result showed that AG treatment obviously attenuated the prolongation of AP in failing myocytes.

### 3.4. Effect of AG on *I*_Ca-L_ Remodeling in Heart Failure

**Figure 4:**
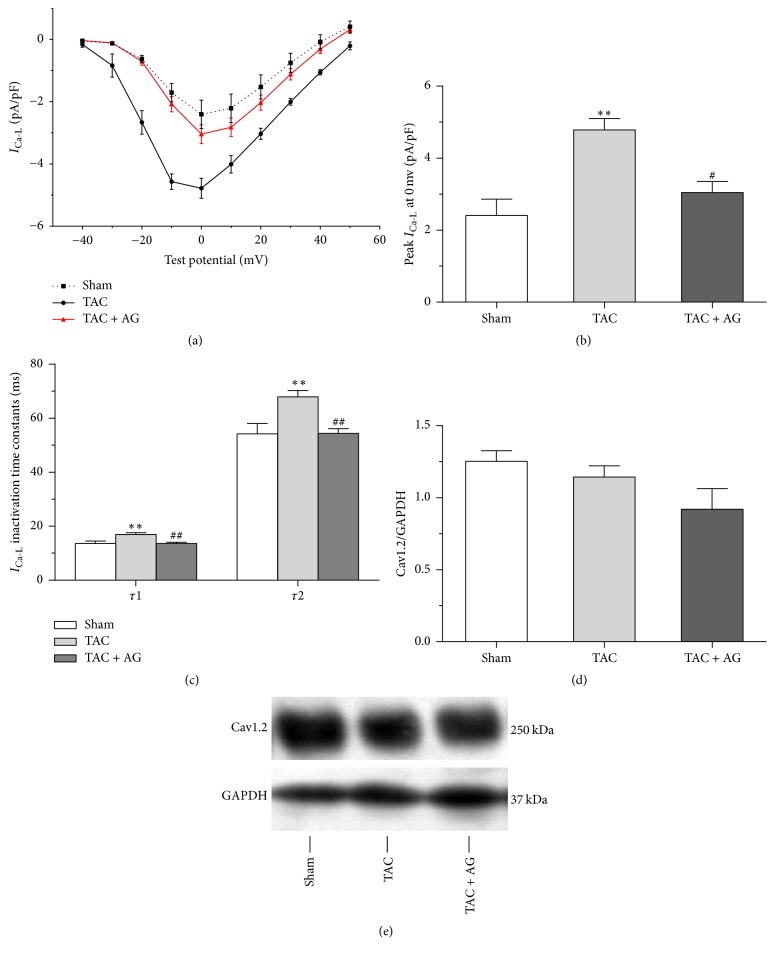
Effect of AG on *I*_Ca-L_ remodeling. (a) The *I*-*V* relationship from groups (*n* = 6). (b) The peak current densities at 0 mV from each group (*n* = 6). (c) *I*_Ca-L_ inactivation fast (*τ*1) and slow (*τ*2) time constants from each group (*n* = 6). (d) Quantification of Cav1.2 expression levels (*n* = 3). (e) Representative western blots. ^*∗∗*^*P* < 0.01 versus sham group; ^#^*P* < 0.05 versus TAC group; ^##^*P* < 0.01 versus TAC group.

*I*_Ca-L_ in myocytes of the three groups was examined to explore the mechanisms responsible for the observed changes in the APD. The peak *I*_Ca-L_ density in myocytes was higher in the TAC group than in the sham group (4.78 ± 0.32 pA/pF versus 2.41 ± 0.46 pA/pF, *P* < 0.01). In contrast, the peak *I*_Ca-L_ density was lower in the TAC + AG group than in the TAC group (3.04 ± 0.30 pA/pF versus 4.78 ± 0.32 pA/pF, *P* < 0.05) (Figures 4(a) and 4(b)).

*I*_Ca-L_ inactivation was significantly slowed at all potentials in myocytes from the TAC group compared with the sham group, which was attenuated in the TAC + AG group. For example, at 0 mV, *τ*1 (16.98 ± 0.59 ms versus 13.56 ± 0.95 ms, *P* < 0.01) and *τ*2 (67.94 ± 2.37 ms versus 54.24 ± 3.79 ms, *P* < 0.01) increased in myocytes from the TAC group compared with the sham group. In contrast, *τ*1 (13.58 ± 0.44 ms versus 16.98 ± 0.59 ms, *P* < 0.01) and *τ*2 (54.33 ± 1.78 ms versus 67.94 ± 2.37, *P* < 0.01) decreased in myocytes from the TAC + AG group compared with the TAC group (Figure 4(c)). However, the expression of L-type calcium channel alpha 1C subunit (Cav1.2) was similar between groups (Figures 4(d) and 4(e)).

### 3.5. Effect of AG on *I*_Ca-L_ Modulation Relative Protein Kinase in Heart Failure

**Figure 5:**
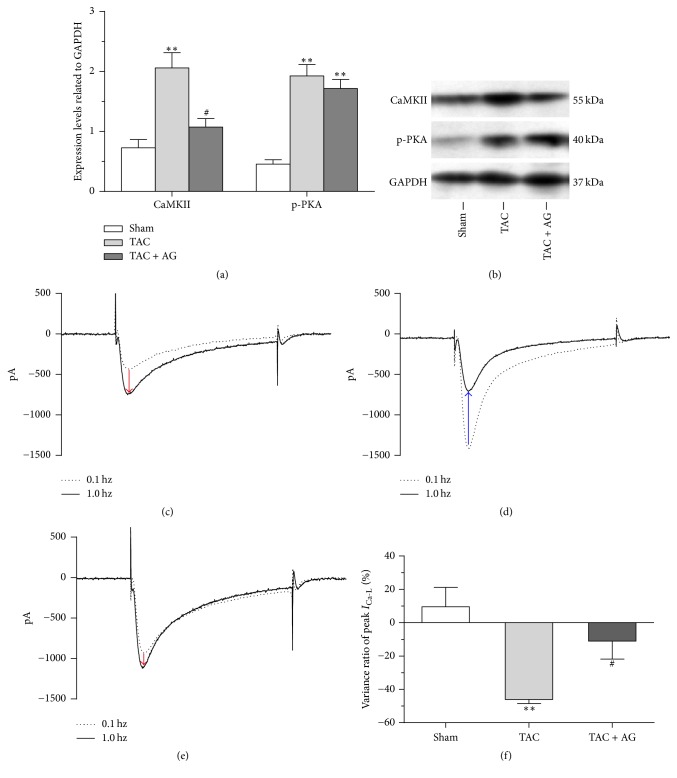
Effect of AG on *I*_Ca-L_ modulation relative protein kinase. (a) Quantification of CaMKII and p-PKA expression levels (*n* = 3). (b) Representative western blots. ((c)–(e)) Typical *I*_Ca-L_ traces at 0.1 Hz and 1.0 Hz from the sham (c), TAC (d), and TAC + AG groups (e). (f) The variance ratio of peak *I*_Ca-L_ between 0.1 Hz and 1.0 Hz from each group (*n* = 6). ^*∗∗*^*P* < 0.01 versus sham group, ^#^*P* < 0.05 versus TAC group; red arrow and blue arrow indicate positive and negative frequency-dependent *I*_Ca-L_ facilitation, respectively.

Two major modulation relative protein kinases, CaMKII and phosphorylated PKA (p-PKA), were assessed in each group. The expression levels of CaMKII (2.057 ± 0.254 versus 0.726 ± 0.136, CaMKII/GAPDH, *P* < 0.01) and p-PKA (1.929 ± 0.189 versus 0.460 ± 0.072, p-PKA/GAPDH, *P* < 0.01) both increased in hearts from the TAC group compared with the sham group. The expression level of CaMKII in the TAC + AG group decreased compared with the TAC group (1.075 ± 0.140 versus 2.057 ± 0.254, CaMKII/GAPDH, *P* < 0.05). However, there was no difference of p-PKA expression between the TAC group and the TAC + AG group (Figures 5(a) and 5(b)).

To validate the changes of CaMKII activation in myocytes, the frequency-dependent *I*_Ca-L_ facilitation was measured. The difference between 0.1 Hz and 1.0 Hz current trace at 0 mV indicated that *I*_Ca-L_ facilitation was significantly blunted in myocytes from the TAC group, which was attenuated by AG treatment (Figures 5(c)–5(e)). The variance ratio of peak *I*_Ca-L_ between 0.1 Hz and 1.0 Hz in myocytes from the TAC group was more negative compared with the sham group (−46.03 ± 2.47% versus 9.60 ± 11.56%, *P* < 0.01). In contrast, the variance ratio of the TAC + AG group was more positive (−11.08 ± 10.77% versus −46.03 ± 2.47%, *P* < 0.05) (Figure 5(f)).

## 4. Discussion

*Astragalus* is a frequently used “Qi-invigorating” herbal medicine to treat HF in Traditional Chinese Medicine (TCM). AG is produced from high-grade authentic raw herb sources of* Astragalus* using a full composition industrial process. Infrared fingerprint quality standard system was adopted to monitor the overall manufacturing process of AG to ensure most decoction ingredients are transferred while achieving high levels of stability and clinical effectiveness. A clinical study has shown the dose-effect relationship of AG in improving the quality of life in patients with chronic heart failure [9]. Several studies reported that the ingredients from* Astragalus* showed effect of cardiac protection during ischemia injury and heart failure [4, 5, 8]. Consistent with those previous findings, our results confirmed that AG protects systolic and diastolic cardiac function in HF mouse model.

In this study, the heart failure mice model was established and the electrical remodeling* in vivo* and* in vitro* was evaluated. The failing heart manifested distinct electrical remodeling including the prolongation of repolarization time and the altered *I*_Ca-L_ kinetics. T-wave alterations and action potential prolongation are common features in experimental models of heart failure as well as in patients with heart failure and are regarded as the predictors of sudden cardiac death [2]. *I*_Ca-L_ is an important inward current in maintaining the membrane potential during the plateau of AP. In the failing heart, the altered *I*_Ca-L_ kinetics usually accounts for the AP prolongation and triggered activity which lead to arrhythmia [10].

Zhao and his colleagues reported that Astragaloside IV, an active ingredient of* Astragalus* and major component of AG, depressed the peak of *I*_Ca-L_ in normal ventricular myocytes from guinea pig, which coincided with our results in *I*_Ca-L_ [12]. Interestingly, Zhao et al. found that Astragaloside IV induced prolonged APD in normal myocytes by inhibition of K^+^ current, as we found AG treatment shortened the APD of failing myocytes. This suggested that the effects of* Astragalus* ingredients on APD under physiological or pathological conditions were different. Although many discrepancies exist regarding the specific ionic and molecular processes occurring in heart failure, studies in animal models and in humans with heart failure have revealed that HF-associated APD prolongation resulted from functional upregulation of inward calcium current and changes in Ca^2+^ current inactivation [10, 13, 14]. In failing myocytes, APD prolongation may prolong calcium channel opening and increase the risk of Ca^2+^ overload which could contribute to arrhythmias [3]. These evidences lead to a view that Ca^2+^ current may be more important than other currents in APD prolongation and arrhythmias under HF. To our knowledge, our current study is the first attempt to evaluation of the effects of AG upon *I*_Ca-L_ of ventricular cardiomyocytes from a failing heart. Since the inhibition of K^+^ current involved in the APD prolongation under physiological condition, our results suggested the AG-induced inhibition of Ca^2+^ current played an important role in the recovery of pathological APD prolongation of failing hearts.

The mechanism underlying the altered *I*_Ca-L_ kinetics in failing hearts is multilevel and multifactorial [15, 16]. Although some investigators found that Cav1.2 expression contribute to electrical remodeling in HF [17], several studies have grown supporting a critical role of protein kinase in the *I*_Ca-L_ remodeling [18–20]. In this study, we found that both CaMKII and p-PKA expression were elevated in HF hearts with unchanged LTCC expression, supporting the view that altered *I*_Ca-L_ kinetics was linked to the enhanced phosphorylation of protein kinases. Another possible mechanism of *I*_Ca-L_ remodeling with unchanged LTCC expression could be the redistribution of functional LTCC from their canonical location in transversal tubules (TT) to the nonnative crest of the sarcolemma, where their open probability was dramatically increased [17, 21, 22]. Sanchez-Alonso et al. reported that CaMKII-dependent phosphorylation of LTCC was increased specifically in crest microdomains without affecting TT domains [21], consistent with our results of CaMKII upregulation in HF.

In this study, CaMKII downregulation appeared to be an important target of AG treatment on *I*_Ca-L_ remodeling in HF, without change in Cav1.2 expression. Dai et al. reported that Astragalus polysaccharide, an active compound from* Astragalus*, exerted its antihypertrophic action via inhibiting CaMKII signaling [8]. Although CaMKII played an important role in the phosphorylation of *I*_Ca-L_ channel [10, 18], PKA activation also triggers similar changes [19, 23, 24]. To specify the effect of AG, we measured the expression levels of CaMKII and p-PKA. Western blots data revealed that AG treatment induced downregulation of CaMKII, but not p-PKA. Besides that, we evaluated the effect of AG treatment on frequency-dependent *I*_Ca-L_ facilitation in HF myocytes. Evidences have revealed that CaMKII is an important contributor to the frequency-dependent *I*_Ca-L_ facilitation [25, 26]. HF cardiomyocytes showed a negative, instead of positive, frequency-dependent *I*_Ca-L_ facilitation, suggesting already maximal activation of CaMKII [10]. Our results showed that AG treatment protected the positive frequency-dependent *I*_Ca-L_ facilitation in the failing myocytes, further supporting our view that AG inhibited the overactivation of CaMKII in HF. As mentioned above, AG-induced relief of *I*_Ca-L_ remodeling could relate to the nanoscale changes in the location of LTCC. Since the effect of* Astragalus* on the transverse tubule (T-tubule) microdomains or the communication between LTCC and ryanodine receptors (RyRs) has not been reported, further experiments are needed to explore the underlying nanoscale mechanism.

Recent studies have shown that CaMKII inhibition prevents the electrical remodeling in failing heart and is a promising antiarrhythmic target in heart failure [25, 27]. The present study revealed that AG treatment inhibited the overexpression of CaMKII, suggesting CaMKII inhibition was one of the targets in AG treatment of heart failure. Further studies are needed to determine the activation and signaling pathways of CaMKII to elucidate the underlying molecular effect of AG.

## 5. Conclusions

These findings revealed that AG treatment protected the failing heart against electrical remodeling and *I*_Ca-L_ remodeling by downregulating CaMKII.

## Supplementary Material

Table 1 in the supplementary material showed echocardiographic parameters after 4 weeks TAC.
Figure 1 in the supplementary material showed HPLC images of standard Astragaloside IV (a) and AG (b) to guarantee the content of Astragaloside IV in AG.
Figure 2 in the supplementary material showed HPLC images of standard calycosin-7-glucoside (a) and AG (b) to guarantee the cotent of calycosin-7-glucoside in AG.
