# Supplementary material for: Astragalus Granule Prevents Ca2+ Current Remodeling in Heart Failure by the Downregulation of CaMKII
Source: Evid Based Complement Alternat Med. 2017 Aug 10;2017:7517358. doi: 10.1155/2017/7517358 (PMC5569633; doi:10.1155/2017/7517358)
Supplement: Supplementary file 1 — Table 1 in the supplementary material showed echocardiographic parameters after 4 weeks TAC. Figure 1 in the supplementary material showed HPLC images of standard Astragaloside IV (a) and AG (b) to guarantee the content of Astragaloside IV in AG. Figure 2 in the supplementary material showed HPLC images of standard calycosin-7-glucoside (a) and AG (b) to guarantee the cotent of calycosin-7-glucoside in AG. [file 7517358.f1.docx]

Supplementary Materials

**Table 1. Echocardiographic parameters after 4 weeks TAC (mean ± SE)**

|  | sham (n=12) | TAC (n=8) | TAC+AG (n=10) |
| --- | --- | --- | --- |
| Temperature ( C) | 32.36±0.16 | 32.14±0.14 | 32.54±0.14 |
| HR (bpm) | 415±8 | 417±9 | 412±6 |
| EF (%) | 43.73±2.11 | 29.16±3.03^**^ | 39.51±3.53^#^ |
| FS (%) | 21.62±1.24 | 13.72±1.55^**^ | 19.34±1.96^#^ |
| LVIDd (mm) | 4.44±0.06 | 4.67±0.19 | 4.42±0.10 |
| LVIDs (mm) | 3.49±0.09 | 4.04±0.23^*^ | 3.58±0.14^#^ |
| LVVd (μL) | 89.86±2.74 | 102.75±10.88 | 89.49±4.77 |
| LVVs (μL) | 51.02±3.19 | 74.30±10.77 | 54.82±4.95 |
| E (mm/s) | 894.88±25.46 | 775.47±55.03^*^ | 910.25±35.01^#^ |
| Em (mm/s) | 39.35±1.90 | 20.02±2.21^**^ | 34.69±1.65^##^ |
| E/Em | 23.29±1.30 | 40.44±2.80^**^ | 26.66±1.50^#^ |

HR: heart rate; EF: ejection fraction; FS: fractional shortening; LVIDd: left ventricle internal dimensions at diastole; LVIDs: left ventricle internal dimensions at systole; LVVd: left ventricle volume at diastole; LVVs: left ventricle volume at systole; E: early diastolic mitral inflow velocity; Em: early diastolic mitral annular velocity. ^*^*P*<0.05 versus sham group, ^**^*P*<0.01 versus sham group, ^#^*P*<0.05 versus TAC group, ^##^*P*<0.01 versus TAC group.


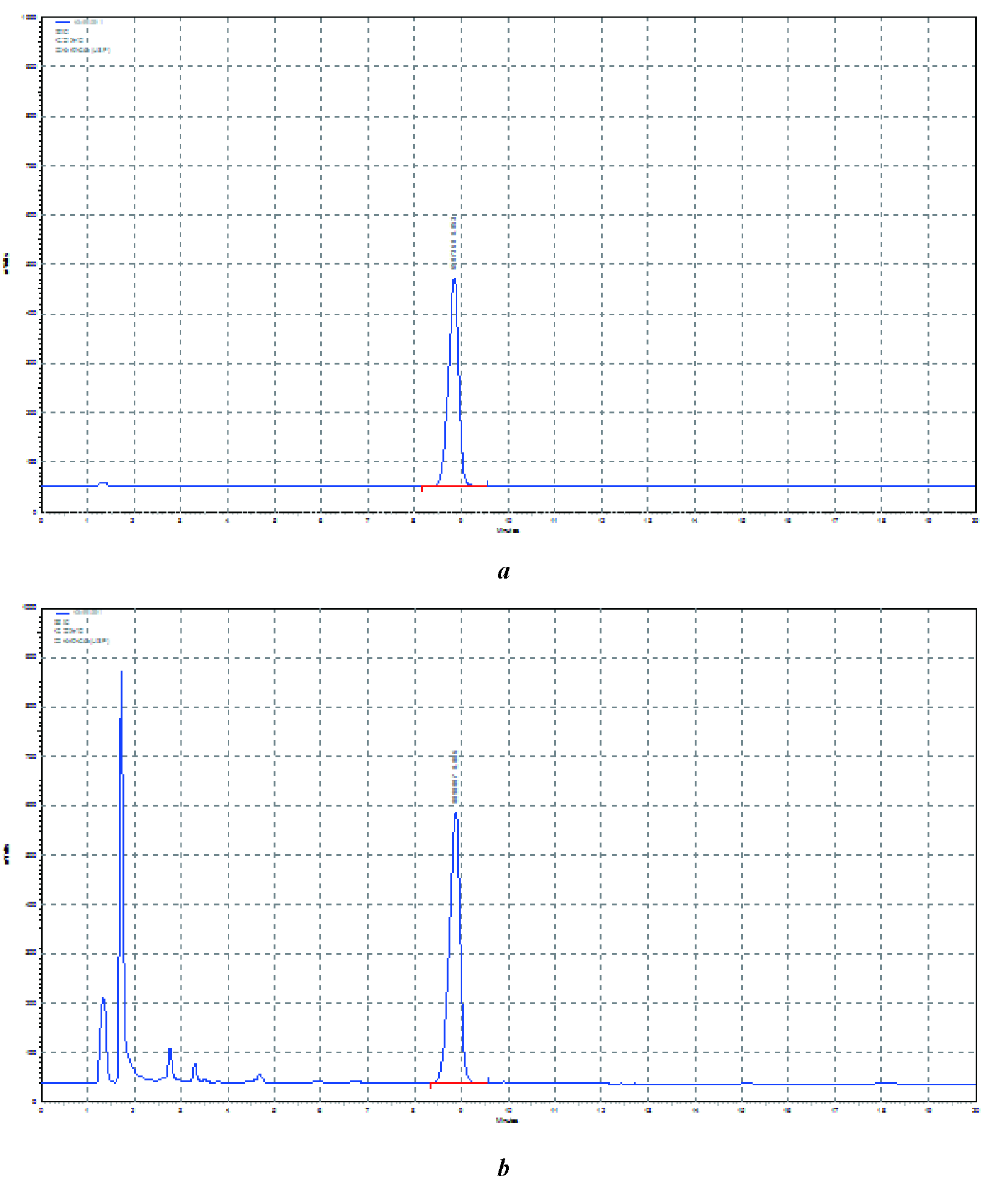


**Figure 1. HPLC images of standard Astragaloside IV (a) and AG (b).**


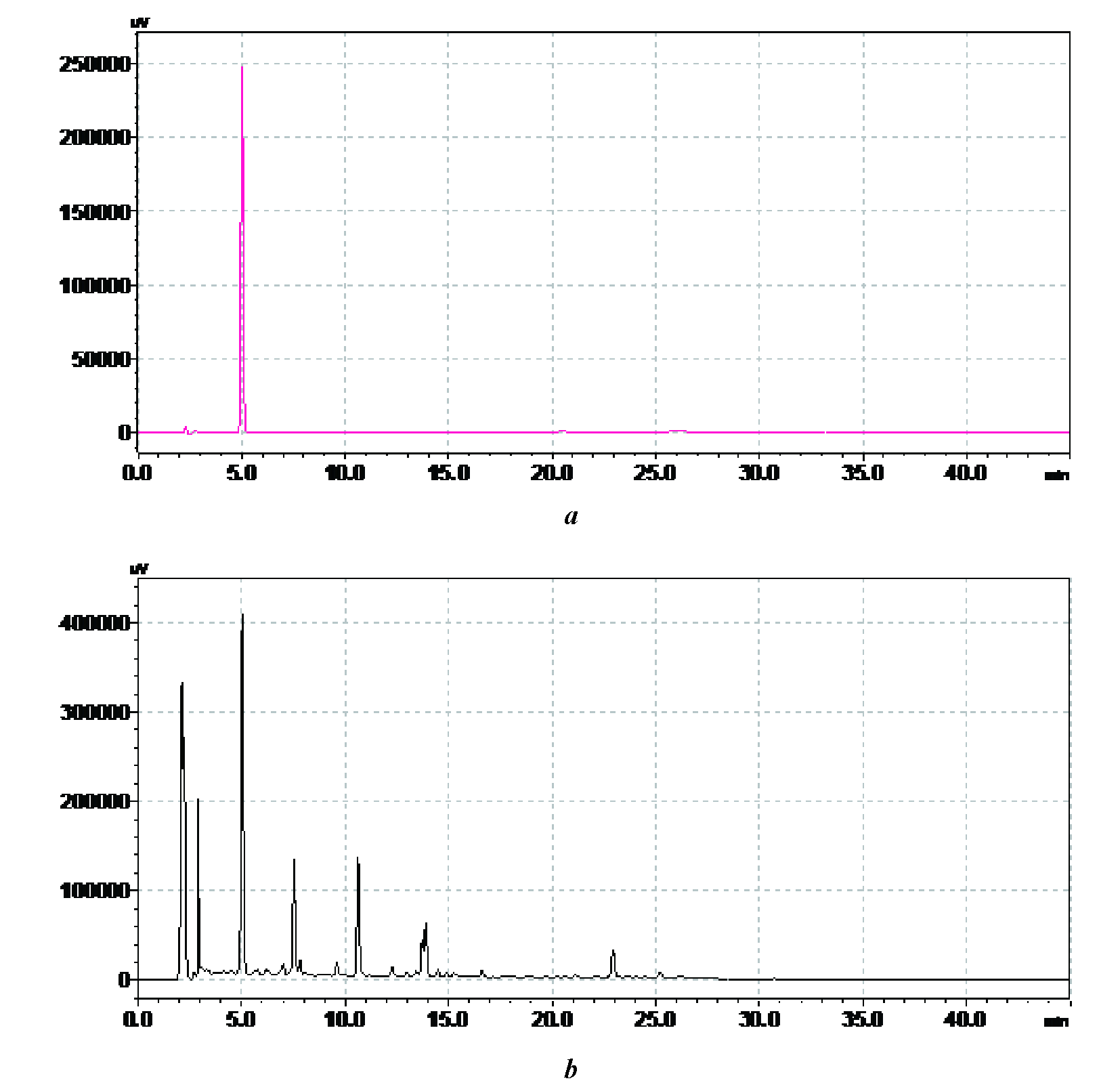


**Figure 2. HPLC images of standard calycosin-7-glucoside (a) and AG (b).**
